# Ischemic Stroke in a SARS-CoV-2-Positive Octagenarian Without Cardiovascular Risk Factors: A Case Report

**DOI:** 10.7759/cureus.23654

**Published:** 2022-03-30

**Authors:** Josef Finsterer

**Affiliations:** 1 Neurology, Neurology and Neurophysiology Center, Vienna, AUT

**Keywords:** bleeding, central nervous system, stroke, covid-19, sars-cov-2

## Abstract

Ischemic and hemorrhagic strokes are increasingly recognized as complications of severe acute respiratory syndrome coronavirus 2 (SARS-CoV-2) infections. The absence of cardiovascular risk factors in SARS-CoV-2-infected patients who suffer a stroke supports a causal relationship as shown in the following report. The patient is an 86-year-old female who developed a mild, brachio-facial, right-sided hemi-symptomatic during a mild infection with SARS-CoV-2. Cerebral magnetic resonance imaging^ ^(MRI) showed a corresponding, small, subacute, ischemic lesion in the left periventricular white matter and a microbleed in the left thalamus. Under dual platelet aggregation inhibitor therapy, the neurological deficits regressed almost completely within three weeks. If one considers that classic cardiovascular risk factors were missing, a causal link between the SARS-CoV-2 infection and the stroke becomes likely. Vasculopathy, coagulopathy, and cardiac disease caused by the virus or the immune response against it serve as pathophysiological explanations for the cerebral lesions. It is concluded that ischemic stroke can occur during infection with SARS-CoV-2. There are more arguments for than against a causal relationship between the viral infection and ischemic stroke.

## Introduction

Coronavirus disease 2019 (COVID-19), the plague of modern times, not only affects the lungs but generally all organs, including the central nervous system (CNS) and the peripheral nervous system (PNS). One of the CNS manifestations of COVID-19 is ischemic stroke [1-2]. It may not only occur in adults but even in children and adolescents [3]. The pathophysiology of severe acute respiratory syndrome coronavirus type-2 (SARS-CoV-2)-associated ischemic stroke is only poorly understood, but there are indications that the occurrence of ischemic stroke is related to the immune reaction against SARS-CoV-2 [4]. SARS-CoV-2 infections are associated with a concurrent elevation of pro-inflammatory and pro-coagulation biomarkers, suggesting these factors are involved in the pathophysiology of SARS-CoV-2-associated ischemic stroke. Furthermore, there are indications that SARS-CoV-2 infections can cause vasculitis, endothelialitis, endothelial dysfunction, and thrombocyte dysfunction. Additionally, SARS-CoV-2-associated endocarditis or myocarditis may result in systolic dysfunction or supra-ventricular or ventricular arrhythmias. Here, we present a senescent without cardiovascular risk factors who experienced an ischemic stroke during infection with SARS-CoV-2.

## Case presentation

The patient is an 86-year-old female, height 166 cm, weight 62 kg, who experienced malaise, mild coughing, and fever up to 38 °C one day prior to admission. She woke up at midnight and when heading for the toilet, noticed pulling to the right and repeatedly touching the wall on her right side. When waking up the next morning, the condition was unchanged and she additionally complained about flu-like symptoms and right-sided facial palsy. Her previous history was positive for glaucoma, cataract surgery bilaterally, and osteoporosis. She did not carry any classical cardiovascular risk factors and denied palpitations or pre-syncope prior to the stroke. Her pre-stroke modified Rankin Scale (mRS) was 0. Her current medication included vitamin D (daily), calcium (daily), timolol drops (daily), and ibandronic acid (every three months). She was admitted for a suspected stroke. She was drinking 1.5 l of non-alcoholic fluids per day.

**Figure 1:**
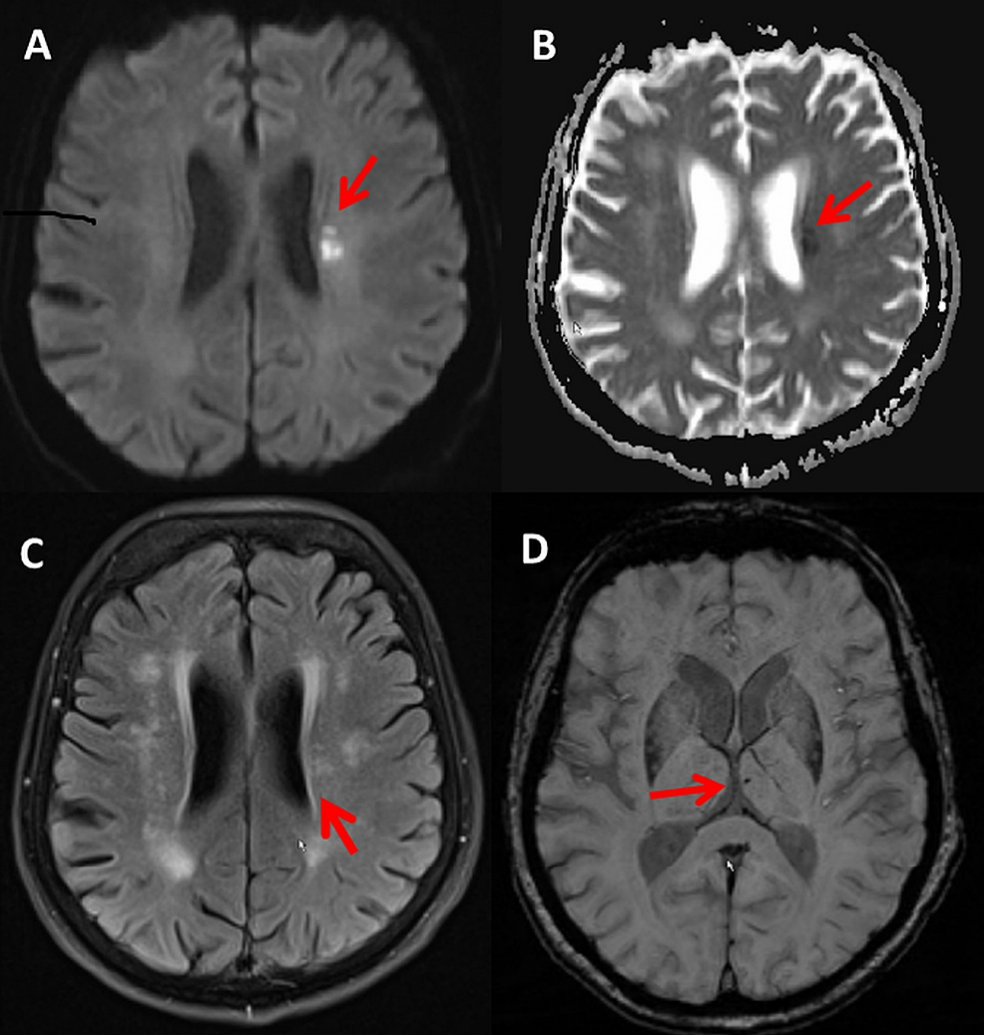
Cerebral MRI showing an acute stroke in the left periventricular region and a microbleed in the left thalamus (A: DWI, B: ADC, C: T2-weighted, D: SWI) DWI: diffusion-weighted imaging; ADC: apparent diffusion coefficient; SWI: susceptibility-weighted imaging

The clinical neurologic exam on admission revealed mild right-sided ptosis, right-sided central facial palsy, spooning (finger flexion) on the right side, mild bradykinesia on the right side, right accentuated tendon reflexes, and an absent Achilles tendon reflex on the left side. Muscle strength was Medical Research Council (MRC) 5 in both arms and legs. No sensory deficit was detected. The Babinski sign was negative bilaterally. The National Institute of Health Stroke Score (NIHSS) was 1. Blood pressure was 142/60 mmHg, electrocardiography (ECG) showed a normo-frequent sinus rhythm. The nasopharyngeal swab test for SARS-CoV-2 by polymerase chain reaction (PCR) was positive on admission. Magnetic resonance imaging (MRI) of the brain revealed a small subacute, ischemic lesion in the left periventricular white matter, which was hyperintense on T2, hyperintense on diffusion-weighted imaging (DWI), and hypointense on apparent diffusion coefficient (ADC) (Figure 1). Additionally, white matter lesions and a microbleed in the left thalamus were found (Figure 1). The carotid ultrasound did not show stenosis or occlusion. Magnetic resonance angiography (MRA) revealed hypoplasia of the left vertebral artery V2 segment. Transthoracic echocardiography showed normally sized cardiac cavities and normal systolic function (ejection fraction: 52%). The patient was discharged without any neurological deficit after 19 days under acetylsalicylic acid (100mg/d), clopidogrel (75mg/d), and rosuvastatin (40mg/d). Rosuvastatin had to be discontinued because of increasing liver transaminases.

## Discussion

The patient is interesting for ischemic stroke during infection with SARS-CoV-2 in the absence of any cardiovascular risk factor. Concerning the etiology of the stroke, several pathophysiological mechanisms can be raised. First, the patient had a transient subclinical episode of atrial fibrillation (AF) leading to intra-cardiac thrombus formation and consecutive thrombo-embolism. An argument in favor of AF is that the prevalence of AF increases with increasing age. An argument against AF, however, is that the index patient’s history was negative for symptoms of AF and that AF had never been recorded on any of her ECGs. Second, the patient had a subclinical hypertensive crisis, leading to focal cerebrovascular vasoconstriction and consecutive ischemic stroke. Arguments against arterial hypertension, however, are that she did not complain about symptoms of arterial hypertension, that never elevated blood pressure values had been measured, and that the patient was never on an anti-hypertensive medication. Third, there was pre-existing microangiopathy, which became accidentally symptomatic. An argument in favor of the microangiopathy hypothesis is that the MRI showed considerable white matter lesions. Arguments against the microangiopathy hypothesis, however, are that the risk factors, arterial hypertension, diabetes, hyperlipidemia, and smoking, were not detectable. The white matter lesions could thus be due to degenerative processes in the context of aging. Fourth, the immune response against SARS-CoV-2 led to hypercoagulability or dysfunctional thrombocytes and thus to intra-atrial or intra-arterial thrombus formation. Arguments in favor of this hypothesis are that thrombus formation is a well-known complication of SARS-CoV-2 infections [5-6] and that embolic stroke has been repeatedly reported in association with SARS-CoV-2 infection [1-2]. Fifth, the infection with SARS-CoV-2 caused cerebral vasculitis, endothelialitis, or endothelial dysfunction, which are suspected to contribute to the pathophysiology of ischemic stroke in COVID-19 patients [7]. Arguments against vasculitis, however, are that the patient had no headache and that the C-reactive protein, blood sedimentation rate, anti-nuclear antibodies (ANA), and anti-cytoplasmic antibodies (ANCA) were normal or negative respectively. Which of these scenarios applies to the index patient remains speculative, but there are arguments in favor and against each of these hypotheses. The absence of any cardiovascular risk factor is a strong argument in favor of SARS-CoV-2 being involved in the pathophysiology of the index patient’s stroke.

The limitations of the study are that no specific tests were carried out to support the pathophysiological effects of the SARS-CoV-2 infection and that no transesophageal echocardiography had been performed.

## Conclusions

This case shows that ischemic stroke can develop during an infection with SARS-CoV-2 and can occur in senescent patients without classical cardiovascular risk factors. Whether the stroke was causally related to the infection remains speculative, but several arguments favor a causal relation.
